# Evolving Robust Gene Regulatory Networks

**DOI:** 10.1371/journal.pone.0116258

**Published:** 2015-01-23

**Authors:** Nasimul Noman, Taku Monjo, Pablo Moscato, Hitoshi Iba

**Affiliations:** 1 The Priority Research Centre for Bioinformatics, Biomarker Discovery and Information-Based Medicine, The University of Newcastle, Newcastle, New South Wales, Australia; 2 School of Electrical Engineering and Computer Science, Faculty of Engineering and Built Environment, The University of Newcastle, Newcastle, New South Wales, Australia; 3 Department of Medical Genome Sciences, Graduate School of Frontier Sciences, The University of Tokyo, Kashiwa, Chiba, Japan; 4 Department of Information and Communication Engineering, Graduate School of Information Science and Technology, The University of Tokyo, Bunkyo, Tokyo, Japan; Leibniz-Institute for Farm Animal Biology (FBN), GERMANY

## Abstract

Design and implementation of robust network modules is essential for construction of complex biological systems through hierarchical assembly of ‘parts’ and ‘devices’. The robustness of gene regulatory networks (GRNs) is ascribed chiefly to the underlying topology. The automatic designing capability of GRN topology that can exhibit robust behavior can dramatically change the current practice in synthetic biology. A recent study shows that Darwinian evolution can gradually develop higher topological robustness. Subsequently, this work presents an evolutionary algorithm that simulates natural evolution in silico, for identifying network topologies that are robust to perturbations. We present a Monte Carlo based method for quantifying topological robustness and designed a fitness approximation approach for efficient calculation of topological robustness which is computationally very intensive. The proposed framework was verified using two classic GRN behaviors: oscillation and bistability, although the framework is generalized for evolving other types of responses. The algorithm identified robust GRN architectures which were verified using different analysis and comparison. Analysis of the results also shed light on the relationship among robustness, cooperativity and complexity. This study also shows that nature has already evolved very robust architectures for its crucial systems; hence simulation of this natural process can be very valuable for designing robust biological systems.

## Introduction

Robustness is a fundamental characteristic that runs throughout every form of life from unicellular bacteria to sophisticated mammals. Therefore, the in-depth understanding of biological robustness has become the prerequisite to complete understanding of biological systems. Naturally, robustness has become the topic of choice to researchers working in a wide range of biological disciplines: from evolutionary biologists [1–3] to computational biologists [4–6], from geneticists [7–9] to immunologists [10, 11], from ecologists [12, 13] to cancer researchers [14, 15]. Numerous studies, undertaken to uncover the features and mechanism of robustness in different biological processes, now enable us to envisage the outline of the bigger picture but many pieces of the puzzle are yet to be discovered to comprehend it.

Today, the commonly accepted perception of biological robustness is the ability of a system to sustain its functionality in the face of perturbation [1, 16]. The source of perturbation could be internal such as mutation and intrinsic noises or external such as environmental changes. These perturbations, whether internal or external, cause to fluctuate the biochemical parameters widely but by virtue of its robustness the system maintains the behavior [17]. Another biological attribute, very closely related to robustness, is redundancy which is the ability of a system to function reliably in spite of one or some of its component failure. Needless to say, such fail-safe mechanism can contribute to the robustness of a system [18], but replicated components demand for additional resources and thus can reduce the performance of the system [19]. Therefore, throughout this work, we adopted the former definition of robustness.

Because of the hierarchical organization of the biological systems, robustness emerges at different levels: genetic level, network level or developmental level. However, the robustness of gene regulatory networks (GRN) is of particular interest because system behavior is believed to be generated by gene interactions [20]. The topology of a regulatory network is attributed for its robustness because these biological systems preserve functionality notwithstanding various alterations in the biochemical parameters by genetic and nongenetic changes. Besides, other research has found that network architecture and composition can contribute dramatically in generating robust network behavior [17] and because of the differences in their wiring, networks with the same number of nodes and interactions may vary drastically in terms of their robustness [21].

Living in the era of synthetic biology we are now aspiring the construction of synthetic gene networks to be fused in biological systems for receiving signal from and sending feedback to the system for therapeutic purposes [22]. But unfortunately, our approach to constructing synthetic networks remains rudimentarily trial and error based [23]. It has been unanimously advocated that *in silico* modeling, optimization and automated design procedure are absolutely necessary to see the awaited outcome from this life engineering paradigm [23, 24]. Addressing this need an array of computational tools and software has been developed to aid the design, simulation and optimization process in synthetic biology. Many of these tools such as Biojade, ProMoT, TikerCell, Jarnac, GenoCAD etc. have become very popular and proven useful for the practitioners. Nevertheless, the issue of robustness in computer aided design of synthetic gene networks lacks sufficient attention.

It has been showed that robustness is an evolvable property—both in experimental studies and computational analyses [25]. Using a computational model Ciliberti *et al.*[21] showed that the meta-graph, in which nodes are networks that differ in their topology, is connected. This connectedness property of this graph makes it traversable and higher robustness achievable from lower robustness through a sequence of small genetic changes in the form of Darwininan evolution [21]. These studies have motivated us to use the computational simulation of evolution for automatic construction of robust gene regulatory network with a particular phenotypic behavior. Since the commencement of post genome era evolutionary algorithms (EAs) remain as a favorable computational approach in reverse engineering of genetic networks from expression data [26, 27]. Recently, a couple of works have appeared that employ evolutionary algorithm for constructing artificial genetic networks automatically [28–31]. A review of these methods can be found in [32]. Because of the inherent relationship of robustness and evolvability and the previous success of EAs in evolution of GRN, we address the problem of automatic construction of robust gene network topology with EAs.

For evolving robust gene network architecture there are two key challenges: i) effectively measuring the robustness of a particular network ii) efficiently calculating the robustness of all networks encountered in the parallel search process. In order to quantify the topological robustness of a gene circuit we need to introduce all possible random perturbation in the system and evaluate the effect. Therefore, measuring the absolute robustness of a network topology becomes a computationally intractable problem. We utilized the Monte Carlo simulation based evaluation method for calculating the robustness of a network structure in a computationally feasible way. Still the robustness measurement of a topology remains computationally very expensive and therefore the fitness calculation of all the network topologies, discovered in the evolutionary search process, becomes computationally very expensive. By exploiting fitness approximation, we designed an evolutionary algorithm that can automatically construct robust network topologies for target functions within reasonable computational budget.

## Results

We employed the proposed method in evolving robust network architecture for two types of behaviors: oscillation and bistability. The existence of a plethora of studies on these two phenomena, both on natural and artificial genetic circuits, helped us to justify the usefulness of the proposed methodology by contrasting the results with present knowledge.

For both types of behavior we tried to evolve genetic circuits by varying the network size and the Hill coefficients. Evolution of the same behavior with different number of genes will help us to characterize the relationship between robustness and circuit complexity. On the other hand, it is relatively easy to change the network behavior by modifying the binding affinity using mutation. So analyzing the effect of Hill coefficient we can understand how robust behavior can be affected by cooperativity.

### Measurement of Robustness

There exists no formal measurement for the robustness of gene regulatory networks. However, we measured the robustness of GRN topology based on the mathematical formulation of biological robustness presented in [19]. In Kitano’s formulation the robustness (*R*) of a system (*S*) with regard to a function (*a*) against a set of perturbation (*P*) is mathematically represented by the following equation

$$R_{a,P}^{s}=\int_{P}\;\psi \left(p\right)D_{a}^{s}\left(p\right)dp$$ (1)

where *ψ*(*p*) is the probability for perturbation ‘*p*’ to take place and *P* is the entire perturbation space. *D*(*p*) is the function that measures to which extent the system preserves its behavior under perturbation (*p*). So *D*(*p*) can be defined as

$$D_{a}^{s}\left(p\right)=\left\{\begin{array}{cc} 0 & \;p\in A\subset P\\ f_{a}\left(p\right)/f_{a}\left(0\right) & \;p\in P-A \end{array}\right.$$ (2)

where *A* is the set of perturbations in which the system fail to retain its target behavior and *f*_*a*_(*p*) and *f*_*a*_(0) are some measurement of the system behavior under perturbation ‘*p*’ and no perturbation ‘0’ respectively. In our implementation we defined *f*_*a*_(*p*) = *f*_*a*_(0) if the GRN topology can retain its functionality according to some criteria. In other words we defined $D_{a}^{s}(p)$ as follows:

$$D_{a}^{s}\left(p\right)=\left\{\begin{array}{cc} 1 & \;\text{if}\;f_{a}\left(p\right)\;\text{satisfies}\;\;\text{some}\;\;\text{criteria}\;\rho \\ 0 & \;otherwise \end{array}\right.$$ (3)

For measuring the robustness of a GRN structure we considered 10,000 random perturbations and realized that by randomly sampling the parameter spaces for that network. Moreover, we assumed that all these perturbations are equiprobable which leaves *ψ*(*p*) = 1 for all *p*. Therefore, the resulting robustness measure for a GRN topology, *G*, becomes

$$R_{a}^{G}=\sum_{i=1}^{10000}D_{a}^{G}\left(p_{i}\right)$$ (4)

where $D_{a}^{G}(p_{i})$ is 1 when the system can retain its behavior under the perturbation ‘*p*_*i*_’ in the defined criteria otherwise zero. All the perturbations *p*_*i*_ are selected randomly. Finally, the score of Eqn (4) is converted into percentage. And all the results presented here are based on this measurement unless mentioned explicitly. We need to define a $D_{a}^{G}(p)$ function for each behavior we want to evolve; more details are presented in Method section.

Kitano’s definition of robustness is very general and can be used in different cases [33]. Using the notion of behavior preservation in the face of perturbation, more formalized frameworks for robustness have been proposed [33, 34]. Rizk *et al.* proposed to evaluate the absolute and relative robustness of a system using the violation degree of temporal logic formula [33]. Donzé *et al.* used signal temporal logic (STL) to define robust satisfaction function in terms of which they defined local robustness and global robustness [34]. Our definition of robustness in this work closely matches with the absolute robustness by Rizk *et al.*[33] and global robustness by Donzé *et al.*[34]. Furthermore, the current definition, based on Kitano’s definition of robustness, is general enough to be adapted to other types of robustness mentioned above.

In this work, we have basically used a Boolean satisfaction criteria in defining the robustness which is analogous to what Donzé and his colleagues have called Boolean semantics [34]. But the above Boolean semantic of robustness in (3) can easily be converted in to quantitative one as follows

$$D_{a}^{s}\left(p\right)=\left\{\begin{array}{cc} |f_{a}\left(0\right)-f_{a}\left(p\right)| & \;\text{if}\;f_{a}\left(p\right)\;\text{satisfies}\;\;\text{some}\;\;\text{criteria}\;\rho \\ 0 & \;otherwise \end{array}\right.$$ (5)

Although throughout this work we have been evolved network topologies using the Boolean semantics of (3), we have done some simulations for evolving network topologies using the quantitative definition of robustness in (5). As we will discuss later, depending on whether Boolean or quantitative semantics is chosen, different network topologies could be evolved.

### Robustness of an oscillatory circuit depends on network size and cooperativity

We searched for robust oscillatory circuits with network size N = 2, 3 and 4 and three levels of cooperativity. Here we considered only positive cooperative binding at three different levels: low (n = 2), medium (n = 3) and high (n = 4). For every combination of network size and cooperativity level, the search procedure was repeated 20 times to ascertain the reliability of proposed stochastic algorithm.

**Figure 1:**
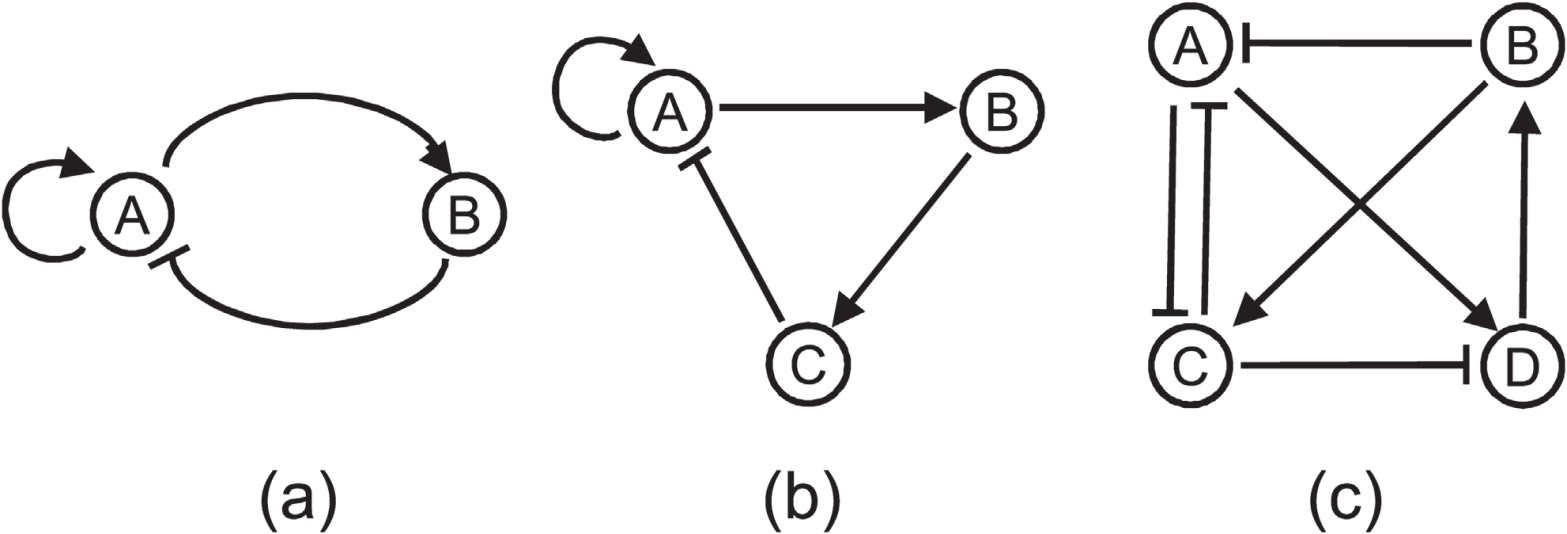
Evolved robust oscillatory network topologies with low cooperativity (n = 2) and different number of genes (N). (a) n = 2, N = 2 (b) n = 2, N = 3, (c) n = 2, N = 4.

Fig. 1 shows the network topologies evolved for different number of genes with low cooperativity. In each of the three studies all 20 evolutionary runs predicted the same network structure as shown in Fig. 1(a), 1(b) and 1(c) respectively. The average robustness (over the 20 repeated runs) of these networks, displayed in Table 1, is found to be increasing with the number of components. From these results it might seem that for the same level of cooperativity if we increase the network complexity then the robustness of the system increases significantly, however, there is an efficiency issue involved which we will discuss in the following section.

**Table 1:** Level of robustness of different evolved network topologies.

Network Properties	Average Robustness	Evolved in evolutionary searches (%)
n = 2, N = 2	00.62%	100%
n = 2, N = 3	04.19%	100%
n = 2, N = 4	85.47%	100%
n = 3, N = 2	00.20%	10%
n = 3, N = 3	80.01%	100%
n = 3, N = 4	99.39%	100%
n = 4, N = 2	00.00%	00%
n = 4, N = 3	94.68%	100%
n = 4, N = 4	99.95%	100%

**Figure 2:**
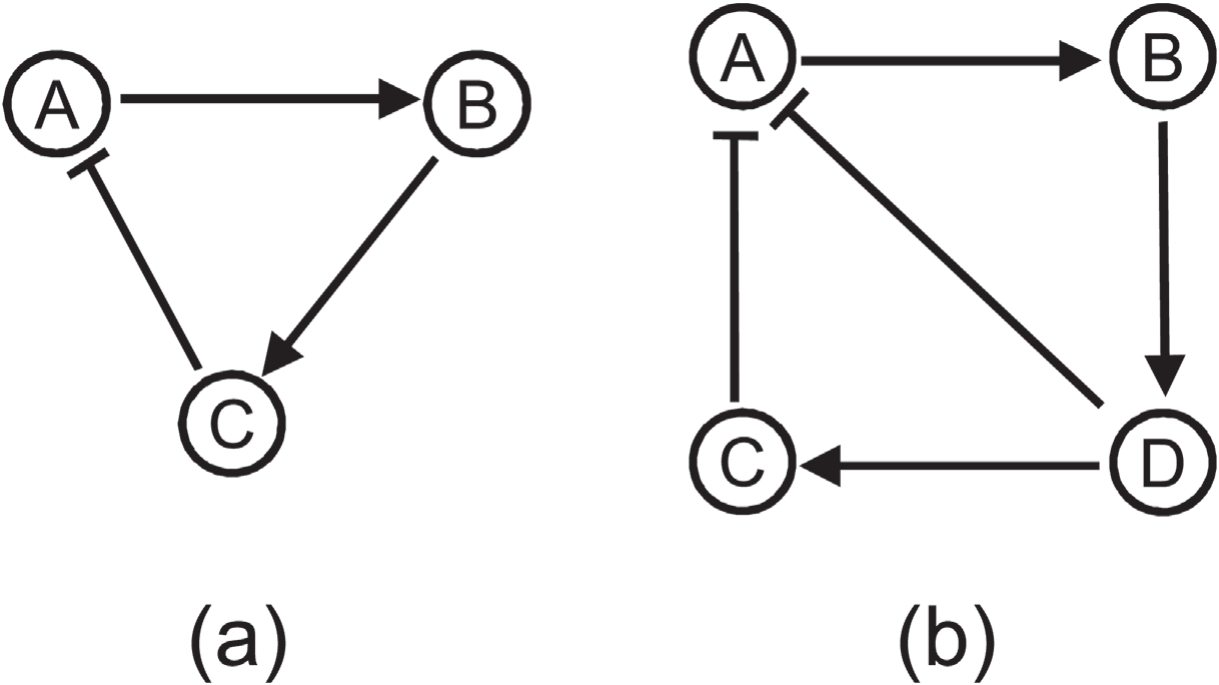
Evolved robust oscillatory network topologies with medium (n = 2) and high (n = 3) cooperativity. (a) n = 3 or n = 4, N = 3 (b) n = 3 or n = 4, N = 4.

Next, we repeated the same set of experiments with medium (n = 3) and higher cooperativity (n = 4). The same topological structures evolved for networks with N = 3 and N = 4 genes, respectively, for both levels of cooperativity (Fig. 2) but exhibited higher level of robustness with increased cooperativity (Table 1). When we increased the cooperativity from n = 3 to n = 4, for 3 gene network the improvement in robustness was significant (from 80.01% to 94.68%) but for 4 gene network the improvement (from 99.39% to 99.95%) was nominal perhaps because of saturation. And in every evolutionary run of our algorithm the same structure was predicted in respective studies. With medium cooperativity (n = 3) and 2 genes we found a network in only 2 runs out of 20 with a robustness score of 0.20%. In both runs the predicted structure was the same as the topology we found with lower cooperativity (Fig. 1(a)) and when the cooperativity was further increased no oscillating network topology with two genes was detected at all. So the robustness of the oscillatory network with two genes decreased with increased cooperativity. This result was particularly opposite to what we observed in other network sizes in which with increased cooperativity robustness of a particular topology increased. However, the oscillation with only two genes is itself very unstable, perhaps therefore it did not generalize. Besides, with increased component numbers (i.e. more genes) the system can exhibit higher robustness. Nevertheless, another observation from this set of experiments is that an oscillatory system can exhibit higher robustness with increased component numbers (i.e. more genes).

### Robustness correlate with cooperativity rather complexity

Earlier results with computational study reported that tightly connected networks are more robust than the sparsely connected networks [35]. However, recent computational modeling suggests sparser networks have some advantage in term of cost of complexity in evolving robustness [36]. Examining the evolved networks, we also observe that almost all the networks predicted by our algorithm are sparse.

We analyzed the topological complexity of all evolved networks in Table 2 by calculating *c*: the connectivity density (*c* = *I*/*N* ^2^) and *K*: the average number of regulators per gene (*K* = *I*/*N*), where *I* is the number of interactions in the network. From Table 2, it is found that for every network the average number of regulators per gene is less than 2. The real gene networks observed in various organisms such as *Escherichia coli*, yeast, *Arabidopsis*, *Drosophila*, sea urchin which have very different number of genes, phylogeny and complexity, are very robust. Interestingly all of them have less than 2 number of transcriptional regulators in these networks [36]. So our network prediction algorithm, that mimics the natural evolution, predicted network topologies with characteristics prevalent in natural gene networks.

**Table 2:** Complexity of the network topologies inferred by the proposed algorithm.

Network Properties	Interactions (I)	c	K
n = 2, N = 2	3	0.75	1.50
n = 2, N = 3	4	0.44	1.33
n = 2, N = 4	7	0.44	1.75
n = 3, N = 2	3	0.75	1.50
n = 3, N = 3	3	0.33	1.00
n = 3, N = 4	5	0.31	1.25
n = 4, N = 2	-	-	-
n = 4, N = 3	3	0.33	1.00
n = 4, N = 4	5	0.31	1.25

One of the observations presented in previous section is that for the same level of cooperativity, the robustness increases with complexity (i.e. number of genes). However, if we take a look in Table 2, the network {n = 2, N = 4} is much more complex than network {n = 3, N = 3}. Although their level or robustness is more or less similar, the network with three components is more efficient in terms of its resource demand compared to the other network. Therefore, it is expected that the natural selection will always prefer the network with 3 genes over the other. Essentially we observe the three gene topology in several natural networks but to the best of our knowledge no reporting of the topology with {n = 2, N = 4} in natural networks. Moreover, we have seen that just increasing the cooperativity level we can have higher robustness for the same network topology. If we compare the gain in robustness for increasing complexity and increasing cooperativity then we can see that for increasing cooperativity we can achieve higher robustness at less cost in terms of resource demand. The positive correlation between robustness and cooperativity has also been reported in other studies [37]. Therefore, we speculate that cooperativity played an important role in evolving robust but sparser hence efficient networks.

### Nature has evolved the most robust topologies

The structures of all oscillators evolved in our algorithm are either already known topologies for oscillation or constructed with motifs which contribute to oscillation. For example, the oscillator with *n* = 2, *N* = 2 (Fig. 1(a)) is the well-recognized amplified negative feedback oscillator which has been realized in different studies [38, 39]. The oscillator topology evolved with *n* = 2, *N* = 3 (Fig. 1(b)) uses the positive feedback and a negative feedback with delay --- two crucial components for oscillation [40] and this topology has been implemented *in vitro* as well [41]. The network with *n* = 2, *N* = 4 (Fig. 1(c)) has positive feedback (*A* ⊣ *C* ⊣ *A*) and two coupled delayed negative feedback loops (*A* → *D* → *B* ⊣ *A* and *A* → *D* → *B* → *C* ⊣ *A*). All these components are known motifs which either generate oscillating behavior or increase robustness of oscillation [40].

The oscillatory topologies evolved with higher cooperativities (*n* = 3 and *n* = 4), exhibited higher robustness and sparser architectures. The evolved network with three components (Fig. 2(a)) is very well known delayed negative feedback architecture for oscillation [40] and the four component circuit (Fig. 2(b)) contains two coupled negative feedback loops (*A* → *B* → *D* ⊣ *A* and *A* → *B* → *D* → *C* ⊣ *A*). In nature we can find several examples of the three component delayed negative-feedback loop: oscillation of p53 in response to ionizing radiation [42], oscillation of nuclear factor-*κ*B in response to stimulation by tumour necrosis factor [43]. The mammalian circadian clock circuit is known to be very robust to genetic perturbations [44]. This circuit has multiple feedback loops [45] which is the case of the evolved 4 component network topology. These correspondences between the evolved oscillating networks and the oscillators in biological systems ascertain that the proposed methodology is capable of evolving robust oscillators that are found in nature. Additionally, it also indicates that nature has evolved the most robust architectures for its crucial systems.

### Evolved topologies are more robust compared to others

Because of their crucial role in circadian systems and appearance in virtually every area of science, oscillators are most widely studied as a single biological process. Consequently, we know a wide spectrum of gene circuits that exhibit different types of oscillations. In order to contrast the robustness of the topologies evolved by our algorithm with that of other known oscillatory topologies we quantified their robustness using a Monte Carlo method.

For each topological structure we sampled the parameter spaces for 100,000 times randomly within the given parameter ranges. For each selected parameter set we checked if the given topology is oscillating or not using the fitness measuring criteria (see the methods section). Thus we counted the number of parameter sets that generated oscillation successfully. And we repeated this procedure 10 times and converted the average to percentage as the robustness measure. For comparison purpose we chose the topology of *repressilator* [46] and the nine topologies reported in [40] as oscillating circuits. See S1 Fig. for these network structures and since the topology in Class 1 was the same topology evolved in our algorithm we did not repeat it in our comparison. All these circuits were compared for all three cooperativity levels. We compared them with three gene network topologies evolved by our algorithm.

From Table 3 we see that for every cooperativity level the evolved topologies were most robust in our quantitative measures. The repressilator circuit was the second best in terms of robustness for medium and high level of cooperativity but for low cooperativity it was not oscillating at all. It should be noted that many of the entries in Table 3 became zero because of rounding. Among the different alternative architectures presented by Novàk and Tyson [40], the Class 3a was found to be most robust in all cooperativity levels. However, the measured robustness of Class 3a oscillator was very low compared to that for our evolved oscillators. This comparison ensures that our algorithm was successful in evolving the most robust oscillating topology with 3 genes. Furthermore, the evolved topologies for other network sizes are the best known robust structures for oscillation.

**Table 3:** Comparison of robustness for various oscillating networks with three components.

Network	n = 2	n = 3	n = 4
Repressilator [46]	00.00	70.94	91.43
Novak Class 2a [40]	00.01	00.03	00.03
Novak Class 2b [40]	00.00	00.00	00.00
Novak Class 2c [40]	00.04	00.09	00.07
Novak Class 2d [40]	00.00	00.00	00.00
Novak Class 3a [40]	00.09	16.27	11.91
Novak Class 3b [40]	02.02	07.32	04.79
Novak Class 3c [40]	00.45	00.00	00.00
Novak Class 3d [40]	00.01	00.01	00.00
Evolved with n = 2	03.59	–	–
Evolved with n = 3 or n = 4 †	–	78.73	93.97

† This is the same network structure with Novak Class 1

We also investigated whether the fitness approximation measure used in our evolutionary algorithm was acceptable. We used the independent robustness quantification with the Monte Carlo method mentioned above and the results are presented in Table 4. Comparing the scores in Table 4 with the corresponding scores in Table 1 it can be confirmed that the measures were very similar except for the case *n* = 2, *N* = 2. These similarities between scores validates the use of the proposed fitness approximation method (details in Methods section) for quantifying the GRN robustness in our evolutionary algorithm.

**Table 4:** Verification of robustness measure for the evolved network topologies.

Evolved Network Properties	Robustness (%)
n2, N2	00.04
n2, N3	03.59
n2, N4	84.51
n3, N2	00.00
n3, N3	78.73
n3, N4	99.16
n4, N2	–
n4, N3	93.97
n4, N4	99.87

### PRCs commensurate with the measured robustness

Although a wide range of sensitivity analysis can be performed on limit cycle oscillators [47], phase response curves (PRCs) are most commonly used for circadian clocks. PRC portrays the magnitude of the time-dependent sensitivity in response to a perturbation given to the oscillatory system [48]. Since the complete sensitivity analysis for all evolved oscillators is beyond the scope and purpose of this study, we present the phase response analysis of the evolved oscillators as an indicator of their ability to robustly entrain to the environment cycles.

**Figure 3:**
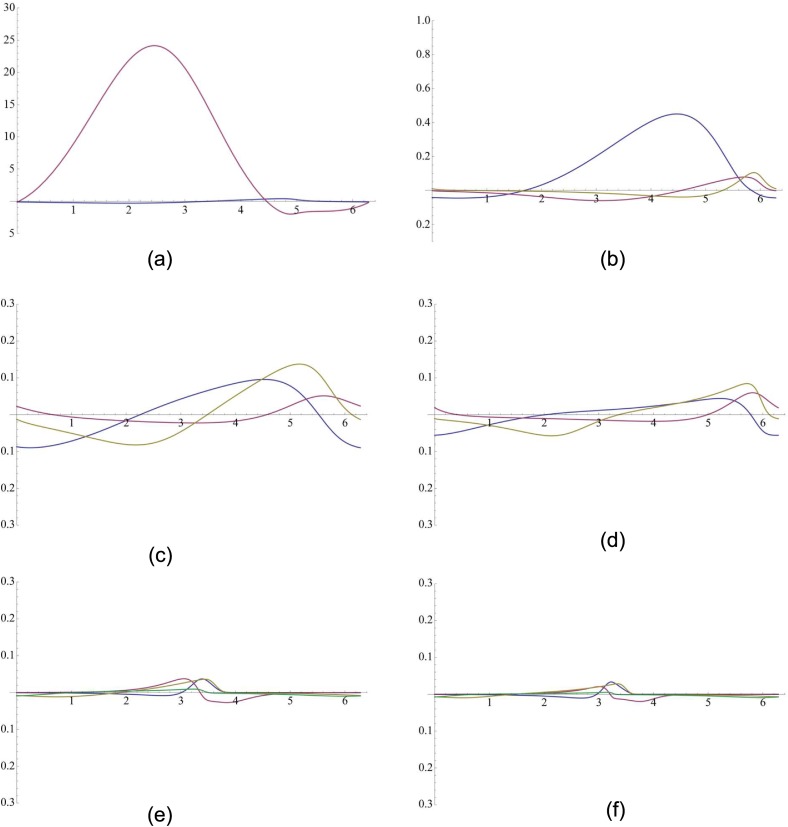
Phase Response curves for different oscillating circuits. The X axis represents the phase (*θ*) and Y axis represents the phase shifts (Δ*θ*). (a) n = 2, N = 2 (b) n = 2, N = 3 (c) n = 3, N = 3 (d) n = 4, N = 3 (e) n = 3, N = 4 (f) n = 4, N = 4.

For each network topology, we randomly chose a parameter set that provides stable oscillatory behavior and then we calculated PRC from that oscillator model. The PRCs for different evolved oscillators are shown in Fig. 3. The PRCs in Fig. 3(c) and 3(d) were calculated from the same parameter set except *n* = 3 and *n* = 4 was used respectively. And the same was done for PRC pairs in Fig. 3(e) and 3(f) respectively. If we compare PRCs for *n* = 2, *N* = 2 and *n* = 2, *N* = 3, we can see that the topology with three genes was less sensitive as we have observed in our measured robustness. And for PRCs in Fig. 3(c) and 3(d) (Fig. 3(e) and 3(f)) we see that the network model with higher cooperativity is more robust which also has been observed in our evolved topologies. From that perspective, it can be stated that the PRCs presented in Fig. 3 indicate that there is a close correspondence between the sensitivities of these evolved topologies and our measured robustness.

### Robustness measured using Boolean and quantitative semantics could be different

We have already pointed out that both Boolean and quantitative semantics can be used to measure robustness. However, the Boolean semantics does not differentiate among the deviations in behavior in response to perturbations; therefore, this could assign a higher robustness score to a topology that actually has greater accumulated deviations from the target behavior in the face of perturbation. In this sense, the Boolean semantics gives a more qualitative measure of robustness which is a valid assumption in many situations. Since the evolutionary algorithm selects topologies based on their fitness score, obviously different topologies can be evolved if the quantitative measure is used instead of the Boolean one. In order to investigate the effect of quantitative semantics, we evolved the oscillating GRN topologies using the measure of (5) instead of (3) with *n* = 2 and *N* = 2, 3, 4. The experimental setup was the same as before and the summary of the results are presented in Table 5.

**Table 5:** Robustness of different network topologies evolved with quantitative measure of (5).

Network Properties	Average Robustness Score	Evolved in evolutionary searches (%)
n = 2, N = 2	98.5903	100%
n = 2, N = 3 −1 (Fig. 4(a))	2385.2154	40%
n = 2, N = 3 −2 (Fig. 4(b))	2390.6491	35%
n = 2, N = 3 −3 (Fig. 4(c))	2364.6971	25%
n = 2, N = 4	41387.6378	100%

**Note**: Here robustness is not measured in percentage.

**Figure 4:**
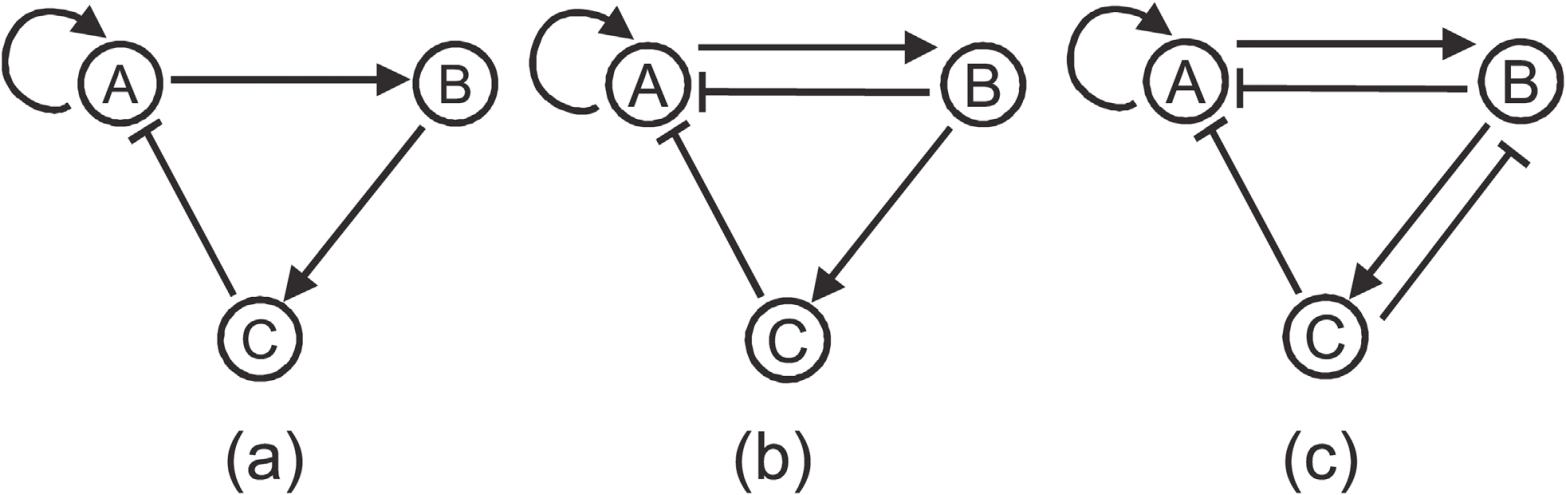
Evolved robust oscillatory network topologies with low (n = 2) cooperativity and N = 3 using the quantitative measure of robustness in (5). (a) Evolved in 40% run (b) Evolved in 35% run (c) Evolved in 25% run.

For *N* = 2 or *N* = 4 the topologies evolved with quantitative semantics were the same as those evolved with Boolean semantics, i.e. topologies in Fig. 1(a) and 1(c) respectively. And all the evolutionary runs evolved the same topology. However, in case of *N* = 3 some of the evolutionary runs evolved the same topology evolved with the Boolean semantics but in other evolutionary runs two other topologies were evolved as shown in Fig. 4. If we check the average robustness scores (in Table 5) for these alternate topologies with *N* = 3 then we find that those are not very different and all the regulations are same except one or two additional regulations appeared in other two topologies. However, as we discussed earlier, if we take the cost of complexity in calculating robustness, the topology evolved with Boolean semantics would be more efficient. Nevertheless, from these observations, it can be deduced that based on how we measure the robustness alternate topologies might evolve.

### Robustness of a bistable switch depends on cooperativity rather the component number

In our second set of experiments, we examined the proposed algorithm’s ability in evolving robust bistable networks. Like oscillatory networks, in evolving robust bistable systems we experimented with all combinations of cooperativity levels (*n* = 2, 3, 4) and network sizes (*N* = 2, 3, 4). We performed 20 repetitions of every experiment and calculated the average for each setup. Table 6 summarizes the average robustness (with standard deviation) of different bistable network topologies.

**Table 6:** Robustness of bistable networks with different component numbers (N) and cooperativity levels (n).

	*N* = 2		*N* = 3		*N* = 4	
	Avg	SD	Avg	SD	fig:bistableNet Avg	SD
*n* = 2	47.22	00.74	49.01	00.74	49.38	00.64
*n* = 3	62.43	00.67	62.27	00.58	62.49	00.60
*n* = 4	68.47	00.71	67.69	76.36	67.95	00.65

**Figure 5:**
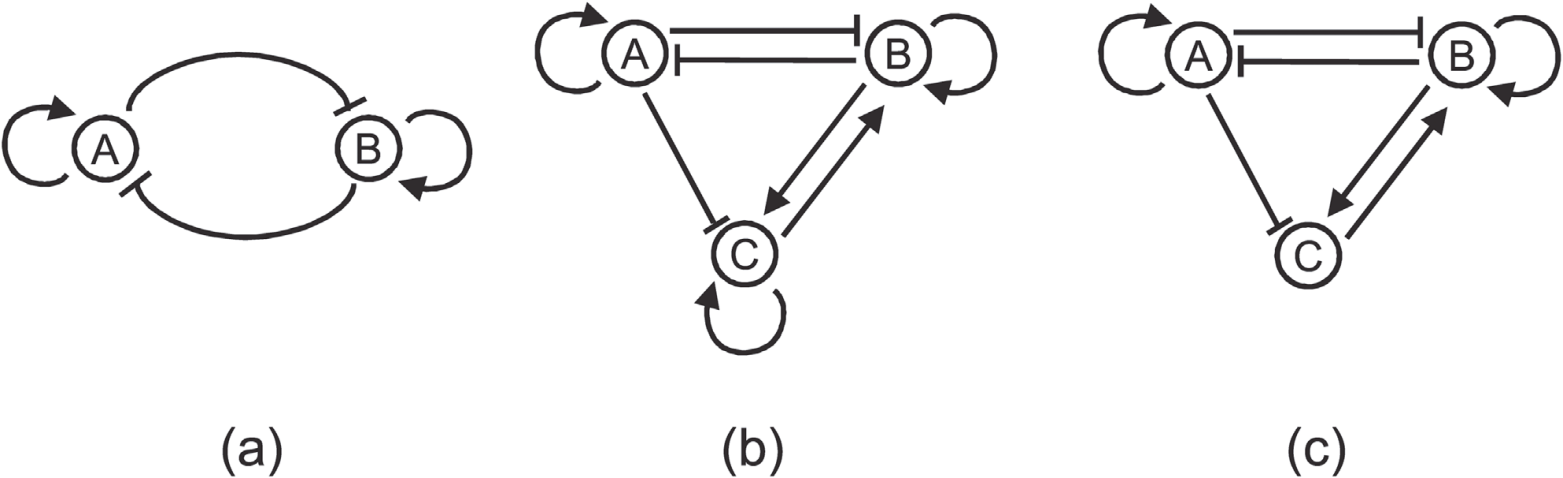
Evolved bistable network topologies. (a) n = 2, 3, 4 and N = 2 (b) n = 2, N = 3 (Net01) (c) n = 3, N = 3 (Net02).

With two genes (*N* = 2) and low cooperativity (*n* = 2), each of the 20 evolutionary runs evolved the same network topology (shown in Fig. 5(a)) for bistablity. For medium (*n* = 3) and higher cooperativity (*n* = 4) levels we received the same topology, as well, in evolutionary run. The bistable network identified in all of our runs is the most well-known architecture for bistable toggle switch with two genes each with positive auto-regulation and mutually inhibiting the other [49]. Moreover, this motif of bistability has been also identified in *E. Coli* [50]. For the low cooperativity level the average robustness of the bistable network was 47.22%. However, as we increased the cooperativity to *n* = 3 the robustness increased to 62.43% and when the cooperativity level was further incrased to *n* = 4 the same circuit exhibited a robustness of 68.47%. Once again, for a completely different network behavior, we observe that cooperativity plays a significant role in determining the robustness of a network topology.

On the contrary, for the same cooperativity level as we increased the network components there were not any significant changes in the robustness levels for higher number of genes. For example, for low cooperativity level when we used three genes, the average robustness increased from (47.22% to 49.01%) and then when we used 4 genes it escalated to 49.38%. More importantly, the algorithm did not converge to the same network topology over different evolutionary runs. For low cooperativity and 3 genes, we found two different network topologies (shown in Fig. 5(b) and 5(c) respectively) of which Net01 and Net02 evolved in 11 and 9 runs respectively. For low cooperativity and 4 genes the algorithm evolved 20 different network topologies in 20 independent runs (results are not shown). Nevertheless, the most important characteristic common in all of these network topologies (both with 3 genes and 4 genes) is that all of them possess the bistability motif of Fig. 5(a). These results indicate that for low cooperativity (n = 2) the robustness achieved by the 2 gene bistability motif of Fig. 5(a) cannot be increased by adding additional genes. The slight improvement observed with increased network components (with 3 or 4 genes and additional regulations) was due to due to some enhanced auto-feedback or negative feedback mechanism added to the system but the improvement in robustness was insignificant so the evolutionary algorithm did not identify any particular structure over different runs. The structures evolved with 3 genes, shown in Fig. 5(b) and 5(c), are almost similar except that one autofeeback regulation in gene C is additional. To further investigate, we compared the two 3 gene networks in Fig. 5 using the same Monte Carlo method we applied for comparing oscillating networks. After analyzing the average robustness scores and standard deviations we could not find any statistically significant difference between these two network topologies in terms of their robustness scores. Although these topologies might offer some robustness enhancement over the two gene network of Fig. 5(a), comparing the overhead of additional components with the robustness improvement, the natural selection will always choose the 2 gene topology over the 3 gene topologies.

For medium level cooperativity (*n* = 3) the algorithm inferred 10 different network topologies for three gene networks (*N* = 3) over 20 repeated runs. The structures are shown in S2 Fig.. And for high level of cooperativity (*n* = 4) 14 different topologies were predicted by the algorithm in 20 evolutionary runs (S3 Fig.). Some of these network structures evolved both with *n* = 3 and *n* = 4. Additionally, in a couple of evolutionary runs the third component of the network was isolated (S2(f) Fig. and S3(j) Fig.) which leaves us with the core 2-gene bistability motif of Fig. 5(a). And for 4 gene networks, both with medium and high cooperitivity, the algorithm predicted 20 different topologies in 20 independent evolutions. More importantly, in case of higher cooperativity (*n* = 3 and *n* = 4) the average robustness scores with more network components (*N* = 3 and *N* = 4) were either less or similar to that with two gene networks (Table 6). Nevertheless, the most important discovery is that all of these network topologies, inferred with *N* = 3, 4 and *n* = 3, 4, possessed the same bistability motif of Fig. 5(a). From all of these observations we hypothesized that the topology of Fig. 5(a) is the most robust network structure for bistability with 2, 3 or 4 genes and adding additional genes or regulation cannot increase its robustness significantly, however, increasing the cooperativity can enhance the robustness of that particular network motif.

## Discussion

Synthetic biology is actually a new approach to biology for designing and constructing novel biological systems that deliver desired behaviors. This life hacking discipline is utilizing engineering principles such as standardization, abstraction, modularity, predictability etc. to assemble biological parts and modules into more complex system in a hierarchical manner. One essential requirement in this process is the robustness of different components to ensure their reliability as well as reusability. However, manual design and implementation of robust modules or systems, which is the most common practice today, is not a trivial task to accomplish. Automated design procedure for robust biological modules will greatly contribute in synthesizing complex living systems thereby take the discipline to its next level.

Current research presents an *in silico* method for automatic design of biological systems or modules that can exhibit robust behavior. Our designed genetic algorithm predicts the topology of a gene regulatory network that is most robust for a particular behavior. Starting from a set of random networks the algorithm explores the search space of network topologies for the optimum one in terms of robustness. We verified the capability of the algorithm in evolving two different networks behaviors, namely oscillation and bistability. We tested the algorithm for different network sizes and levels of cooperativity and most of the times the algorithm identified the robust network structures which are known to exist in nature as well as predicted a few novel structures. Comparing with other natural or artificial topologies the evolved networks were found to be more robust.

The main contribution of this work is to show that it is possible to design a robust GRN topology using the computer simulation of natural evolution. One important component of this process is the robustness measurement of network topologies. Here, we have used a very generalized definition of robustness after Kitano [19]. Our definition of robustness is similar to what the other works have defined as absolute robustness [33] or global robustness [34]. Although, in this work we are using a less formal definition of robustness, comparing with other known topologies it was found that the current definition is useful in estimating the robustness of oscillatory and bistabile networks. However, because of its generality, we can substitute the current definition with some more rigorous definition of robustness or other types of robustness as found in [33, 34], and the evolutionary framework would find the most robust topology in accordance with that definition.

Another contribution of this work is to present an efficient method for quantifying the robustness of a GRN topology based on Monte Carlo method. Since in the evolutionary search we need to estimate robustness of hundreds of alternate GRN topologies, in order to to accelerate the robustness calculation of GRN we proposed a fitness approximation process. The utilization of this fitness approximation method as well as an archive of evaluated topologies significantly helped to speed up the overall search process of our algorithm. The ‘*ApproxThreshold*’ parameter in our fitness evaluation process for GA which actually measures the robustness of a topology, plays an important role in reducing the computational cost of our algorithm. This parameter to be set such that the fitness of the topologies with a robustness value below a threshold will be approximately calculated rather exactly determined. Because of their lower robustness (fitness scores) most of these topologies will not survive in the long run, so approximate estimate of their fitness value suffices. In the beginning of the search, perhaps most of the topology will not exhibit the target behavior at all or will not be very robust to perturbation, hence the approximate fitness estimation will accelerate the search process considerably.

We also utilized an archive for storing every individual evaluated in our GA. This archive saves the computation for reevaluating the same topology. Towards the end, as the search converges, we will encounter the same topology several times and the archiving will save substantial amount of computation. But at the same time the parameter ‘*ReevaluateNet*’ will guard against any accidental poor score received by any potential topology due to approximation or just because of randomness. Unless the target network is expected to be weakly robust this parameter can be set to some small value between 0.10 and 0.20. Consequently, with reasonable choice of these two parameters our algorithm can perform very efficiently in identifying the most robust network topology.

It might be argued that for 2 gene networks the algorithm explored too many individuals whereas we have only 3^4^ possible topologies. Here, we want to mentioned that the evolution of 2-gene networks was just for proof of concept and the most robust topology was actually evolved within the first couple of generations in every case. In order to maintain the uniformity of the algorithm setup we did not change the population size or generation number. Moreover, because of the use of the archive, the algorithm will not repeatedly evaluate the same topology thereby unnecessarily explore the search space. The proposed GA exhibited it superiority in larger search spaces (3^*N*^2^^ where *N* is the number of genes) and found the optimum solution without searching exhaustively.

In our framework we used the GRN model proposed in [51] to represent genetic interactions. Since we need to measure the robustness of different network topologies by examining a large number of system responses as well as the GA will operate with multiple network topologies in parallel, we needed to choose a GRN model that can offer both system details and computational efficiency. A gene network represented using the selected model is realizable in experiments with proper choice of components whilst the model is reasonably efficient to simulate *in silico* for responses. However, the algorithmic framework presented in this work is generalized enough to replace the model with any other suitable modeling approach. Additionally, in this work we evolved robust GRN topology in which we tested the structures for all possible perturbations. However, the method can be equally used for evolving network architectures which are robust to a particular perturbation e.g. by randomly sampling a specific parameter. Although the definition of robustness used in this work is very general, it is possible to use more formalized definition of robustness based on temporal logic and violation degree [33, 34] in the current framework. As a proof of concept, we have briefly experimented with a quantitative definition of robustness. The proposed fitness approximation method makes the robustness calculation computationally feasible and applicable to systems with large number of parameters. By approximating the robustness of less prominent topologies the method makes the estimation of global robustness for hundreds of networks in a reasonable time thus making it scalable for evolution of larger networks. In this work, we justified our claim about evolving the robust GRN topology using two system behaviors: oscillation and bistability. However, the framework is equally applicable for evolving topology for other types of robust responses. In order to evolve a particular type of response we need to define the *f*_*res*_(*p*) and its relation with *f*_*res*_(0). And we can keep the rest of the algorithm unchanged. With an appropriate definition of this behavior measuring function the algorithm can evolve the network topology that can generate the target system behavior robustly.

As discussed above, the proposed approach is generalized enough to evolve network topologies which are robust to a set of parameters. Without any loss of generality we can assume that the initial concentrations of a system are part of its parameter set. Throughout this work we have kept this parameters (the initial concentration) fixed as well as the cooperativity constants and the number of network components. As a proof of concept, we performed some experiments to evolve oscillatory network topologies which are robust to initial concentrations as well. We repeated the experiments for evolving robust network topologies with *n* = 3, 4 and *N* = 3, 4. All the experimental conditions were the same as before except the initial concentrations of network components were chosen randomly from [0.0, 10.0] nM.

In general, if we change the set of parameters or the sensitivity range of a parameter for which we are evolving a robust system, then it is expected that we will find a different topology. That is because the change in the parameter space will result in a different robustness score for a GRN topology and the evolutionary algorithm will perform selection based on that fitness score. However, in our experiment sets we found that the evolved oscillatory systems were exactly the same as in Fig. 2(a) and 2(b) respectively in every evolutionary run. Not only the network topology but their robustness scores were also very similar—the differences in scores were not statistically significant. Although these results are not generalized, in these cases our evolved oscillatory network topologies were robust to initial concentrations chosen from [0.0, 1.0]nM. The general conclusion from these experiments is that the proposed methodology can be applied to evolve systems robust against any set of parameters.

The results derived in this work have also reinforced some of the existing hypotheses regarding how the robustness of GRN has been evolved. One of the most important one is that the cooperativity is of primary importance in evolving robust GRN. Generally, the robustness of a particular topology increases with the cooperativity. For both oscillation and bistability which are two completely different types of behaviors this has been confirmed in our experiments. Results from varying network sizes for the both types of behaviors further supported the positive correlation between robustness and cooperativity. Another observation is that the topological complexity is not correlated with robustness. In general all the evolved network structures were very sparse rather densely connected which verifies the inherent relationship between the sparsity and robustness. However, some improvements in robustness were noticed when additional network components (genes and regulations) were added to the network. But often the enhancements were minor in particular if we take the additional resource requirements into account. These results coincide with recent studies that suggest that sparse GRN topologies deliver higher robustness. Accumulating these observations we conclude that cooperativity rather complexity of a network contributes to its robustness.

Almost all of the network topologies evolved for both oscillation and bistability behavior are known to exist in biological system. Additionally when the evolved topologies were compared with other known topologies—the evolved ones were found to be superior in robustness measure. This finding has two implications. First, for the above two behaviors the proposed methodology was successful to identify GRN topologies with characteristics that the natural GRNs possess. Secondly, nature has evolved very robust network systems for its essential systems like oscillation or bistability. Besides, the proposed method can also provide us with some novel design for robust GRN as it delivered for four gene networks with low cooperativity.

In conclusion, it can be said that the proposed GA with the fitness approximation technique offers a useful framework for automatic designing of robust gene regulatory networks. By simulating the natural evolution in computer it identifies the most robust network topology for a particular behavior. We also presented an efficient and effective mechanism for quantifying robustness for GRN which could be equally applied to other types of biological systems. The proposed methodology can be valuable for designing alternate robust system for a target behavior as well as can be utilized for understanding how evolution derived different robust architectures for various systems.

## Materials and Methods

### GRN model

We used a differential equation based model, introduced in [51], to represent gene networks. In this model, a set of coupled differential equations is used to represent gene interactions in the form of inhibitions or activations of the rate of transcription of one species by another. The equations also explicitly include the degradation process and every participating species is represented using a continuous variable. The mathematical representation of the model is

$$\frac{dG_{i}}{dt}=a_{i}\cdot \prod_{j}\left(\frac{{Ki}_{j}^{n_{j}}}{I_{j}^{n_{j}}+{Ki}_{j}^{n_{j}}}\right)\cdot \prod_{k}\left(\frac{A_{k}^{n_{k}}}{A_{k}^{n_{k}}+{Ka}_{k}^{n_{k}}}\right)-b_{i}\cdot G_{i}$$ (6)

where, *I*_*j*_ and *A*_*k*_ represent inhibitors and activators, respectively, which act independently of each other. *a*_*i*_ denotes the basal rate of transcription and *b*_*i*_ indicates the rate of degradation. *Ki*_*j*_ and *Ka*_*k*_ represent concentrations at which the effect of the inhibitor and activator, respectively, is half of its saturating value. *n*_*j*_ and *n*_*k*_ describe the degree of cooperativity. They work like Hill coefficients in enzyme kinetics and regulate the sigmoidicity of the curve. The total number of kinetic parameters in this model is 2(*N*+*I*) where *N* is the number of genes, and *I* is the total number of gene interactions.

In our experiments, we chose the number of genes *N* from {2, 3, 4} and set the cooperativity coefficients *n* to low (*n* = 2), medium (*n* = 3) or high (*n* = 4). For any particular gene network, we assumed all cooperative coefficients to be equal (i.e. *n*_*j*_ = *n*_*k*_). In order to quantify the robustness of a particular network topology, we varied all other parameters by setting them randomly within the biologically feasible ranges. The parameter ranges we used are as follows: *a*_*i*_ ∈ [10.0, 100.0], *b*_*i*_ ∈ [0.02, 0.15], *Ki*_*j*_ & *Ka*_*k*_ ∈ [10.0, 100.0].

### Algorithm

We used a genetic algorithm (GA) for evolving robust network topologies starting from random network architectures. Mimicking the natural evolution, GA utilizes a parallel search procedure in which each search point, known as individual, represents a solution for the problem. The fitness score, assigned to each individual, represents the quality of the individual in solving the problem. A better solution receives higher fitness score than a poor solution. The current set of solutions, called population, proceeds through the search period entering from one generation to another. In order to create higher quality solutions (offspring), crossover and mutation operators are applied on individuals (parents) selected from current generation based on some criteria. Higher quality offspring replaces individuals from the current generation thus creating a new generation of population. Because of the selection pressure from the replacement strategy and parent selection, GA search proceeds towards the optimum solution exploring the search space.

In our problem each network topology is represented as an individual. We used a *N* × *N* matrix *M* to represent a network topology where *M*_*ij*_ represented the regulation type from gene *j* towards gene *i*. *M*_*ij*_ can take one of the values from {1, −1, 0} where 1 represents activation, −1 represents repression and 0 represents no interaction.

For choosing parent we applied the tournament selection strategy with a tournament size of 5. The chosen parents participated in the crossover operation which bred the offspring by randomly choosing either a vertical or a horizontal crossover point across the matrix representing each parents. Then the offspring underwent the mutation operation in which the individual entries of the matrix were changed randomly to one of the other two possible values. We applied the crossover and mutation operations with probability 0.5 and 0.15 respectively. For generation alternation 50% worse individuals were replaced by the newly created offspring. The population size was chosen to be 100 and the algorithm iterated for 100 generations before reporting the best result.

### Fitness evaluation

Quantifying the robustness of a network topology is the most computationally expensive part of our algorithm. It can be easily shown that any robustness measure, even with a linear model, is NP-hard [52]. Therefore, we utilized a Mote Carlo method for estimating the robustness of a particular topology. For each topology we randomly sampled the parameter spaces for *a*_*i*_, *b*_*i*_, *Ki*_*k*_ and *Ka*_*k*_ 10,000 times and tested the network behavior for each of these parameter sets. We counted the number of parameter sets for which the network preserved the expected behavior. Then we converted the count into percentage as a measure of robustness of the network topology.

**Figure 6:**
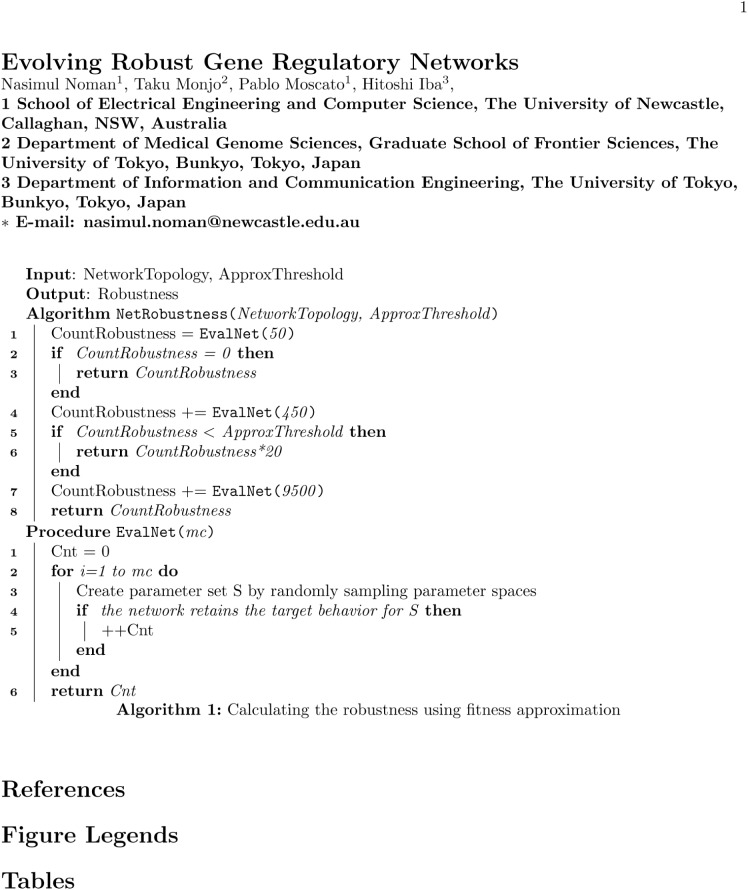
Algorithm for calculating the robustness using fitness approximation.

Even with the Monte Carlo method the fitness estimation becomes very expensive as we need to simulate 10,000 network’s behavior to quantify the robustness of each topology. In order to accelerate the robustness measurement procedure we utilized a fitness approximation technique in our algorithm. A good fitness approximation technique for expensive fitness functions can accelerate the genetic algorithm significantly without compromising its ability to locate the optimum or pseudo optimum solution for the problem [53]. We used a heuristic approach to approximating the network robustness shown in the Algorithm in Fig. 6. The approximation method can help in achieving notable acceleration in robustness calculation process.

In the proposed GA we also utilized an archive to store all fitness evaluated network topologies along with their robustness score. Before we evaluate the fitness score of a network topology using the Algorithm in Fig. 6, first we search in our archive and if the network is found in the archive then we revaluate with the probability *ReevaluateNet*. If the fitness of a topology is reevaluated then we update the archive with the new robustness score for that topology if the score is higher than the stored value. In other words, in case of the fitness reevaluation of a network topology, we always retained the maximum robustness score.

### Measurement of system behavior

As described in ‘Measurement of Robustness’ section, we need to define a *f*_*a*_(*p*) and *f*_*a*_(0) function for each behavior we want to evolve. First, we generated the time series for the topology with random parameter set and from the time series we calculated *f*_*a*_. For oscillation we generated a time series of 2100 minutes starting from an initial value of 1 nM for each gene *G*_*i*_ and assumed the system is oscillating if every gene is oscillating. The initial 300 minutes of the generated time series were allowed for stabilization of time series therefore skipped in our calculation. For each gene we checked the time series within the interval (300 to 2100 mins) and calculated the following function

$$f_{osc}=SD*LC*PN$$ (7)

where *SD* is the standard deviation of the sample points over the considered time interval capped at a maximum value *SD*_*max*_, *LC* evaluates the proximity of a limit cycle by comparing the amplitude of the first and last peaks of the signal sequence and gives a maximum value of 1 in case of a perfect oscillation. *PN* penalizes the overall score in case of damped oscillation otherwise *PN* = 1. Hence, the maximum value that *f*_*osc*_ can return in case of perfect oscillation is *SD*_*max*_. We calculated the average *f*_*osc*_ over all time-series generated by the system and we set the satisfying criteria as *f*_*osc*_ ≥ (*SD*_*max*_)/2.0.

In the other set of experiments for evolving bistability, we simulated the network from 0 to 600 minutes and checked for bistability in *G*_0_ and *G*_1_. We assigned 100nM and 300nM to gene *G*_0_ and *G*_1_ and all other genes (if there is any) were set to 200nM at be beginning of simulation. We selected a system to be bistable, i.e. we set *f*_*bst*_(*p*) = *f*_*bst*_(0), if the following two conditions are met: i) the output fluctuation in *G*_0_ and *G*_1_ is within 0.01 nM in the time interval 400 min to 600 min (i.e. first 400mins were skipped to allow stabilization) and ii) the gene with higher initial value stabilizes at a higher level and the one with lower initial value stabilizes at a lower level. We repeated the process by initializing *G*_0_ and *G*_1_ with the opposite values.

## Supporting Information

S1 Fig Oscillating motifs by Novák and Tyson [40] with 3 components. In these motifs, A → B means ‘A activates B’ and A ⊣ B means ‘A inhibits B’. Note, here we did not discriminate among the roles (e.g. activator, inhibitor, intermediate) played by different components.
(EPS)

S2 Fig Bistable networks evolved with medium cooperativity (*n* = 3) and three network components (*N* = 3). We ordered the networks based on the number of runs in which the topology evolved and it is shown by the number in parenthesis.
(EPS)

S3 Fig Bistable networks evolved with high cooperativity (*n* = 4) and three network components (*N* = 3). We ordered the networks based on the number of runs in which the topology evolved and it is shown by the number in parenthesis.
(EPS)
